# Supplementary material for: N4BP1 negatively regulates NF-κB by binding and inhibiting NEMO oligomerization
Source: Nat Commun. 2021 Mar 2;12:1379. doi: 10.1038/s41467-021-21711-5 (PMC7925594; doi:10.1038/s41467-021-21711-5)
Supplement: Supplementary file 1 — Supplementary Information [file 41467_2021_21711_MOESM1_ESM.pdf]

Supplementary Information

N4BP1 negatively regulates NF-κB by binding and inhibiting NEMO oligomerization

Hexin Shi[1], Lei Sun[1], Ying Wang[1], Aijie Liu[1], Xiaoming Zhan[1], Xiaohong Li[1], Miao Tang[1], Priscilla Anderton[1], Sara Hildebrand[1], Jiexia Quan[1], Sara Ludwig[1], Eva Marie Y. Moresco[1], Bruce Beutler[1]*

[1] Center for the Genetics of Host Defense, University of Texas Southwestern Medical Center, 5323 Harry Hines Boulevard, Dallas, TX 75390-8502, USA.

* Correspondence to: Bruce.Beutler@UTSouthwestern.edu

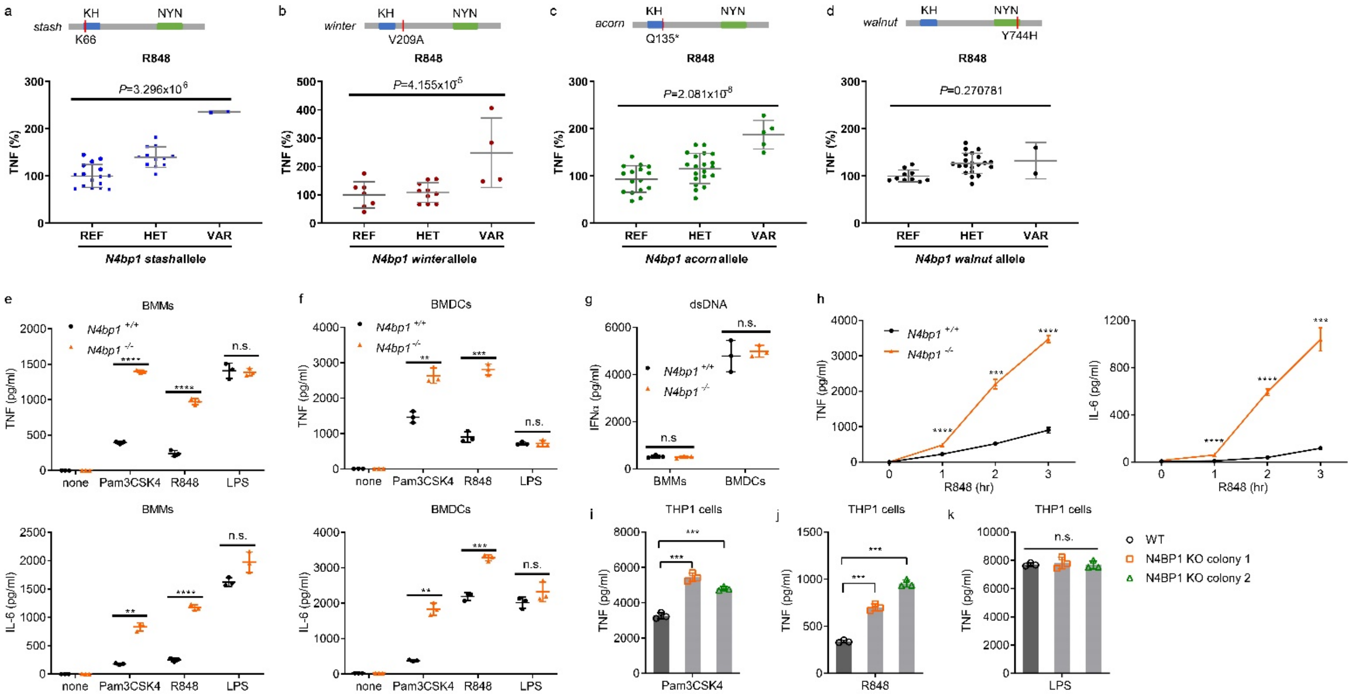

Supplementary Figure 1. Enhanced TRIF-independent TNF and IL-6 production by N4BP1-deficient mouse BMMs, BMDCs and human monocytes. a-d, Mutants in Fig.1b were shown individually with each p value. (**a**) *stash*, (**b**) *winter*, (**c**) *acorn*, (**d**) *walnut.* The *P* values shown in (**a-d**) were calculated using a likelihood ratio test from a generalized linear model [main ref. 41]. **e,f,** TNF (above) or IL-6 (below) concentration in the culture medium of *N4bp1*$^{+/+}$ and *N4bp1*$^{-/-}$ BMMs (*****P*<0.0001, ***P*=0.0025, unpaired, two-tailed Student's *t*-test) (**e**) or BMDCs (***P*=0.0023, ****P*=0.0001 [above]; ***P*=0.0041, ****P*=0.0002 [below], unpaired, two-tailed Student's *t*-test) (**f**) treated with the indicated stimuli. n=3 mice per genotype. **g,** IFNα concentration in the culture medium of *N4bp1*$^{+/+}$ and *N4bp1*$^{-/-}$ BMMs and BMDCs treated with dsDNA. n=3 mice per genotype. **h,** Time course analysis of TNF (left) or IL-6 (right) concentration in the culture medium of *N4bp1*$^{+/+}$ and *N4bp1*$^{-/-}$ peritoneal macrophages treated with R848. n=4 mice per genotype (*****P*<0.0001, ****P*=0.0001 [left], ****P*=0.0003 [right], unpaired, two-tailed Student's *t*-test). **i-k,** TNF concentration in the culture medium of wild-type or *N4BP1*$^{-/-}$ THP1 cells treated with Pam3CSK4 (****P*=0.0004, ****P*=0.0004, unpaired, two-tailed Student's *t*-test) (**i**), R848 (****P*=0.0009, ****P*=0.0003, unpaired, two-tailed Student's *t*-test) (**j**) or LPS (**k**). n=3 independent treatments of 3 cultures split from one cell line (i-k). Each symbol represents an individual mouse (**a-g**) or independent culture (**i-k**). Data are representative of two independent experiments (**e-k**) (mean ± s.d.). *P* values are listed in order from left to right. Source data are provided in the Source Data file.

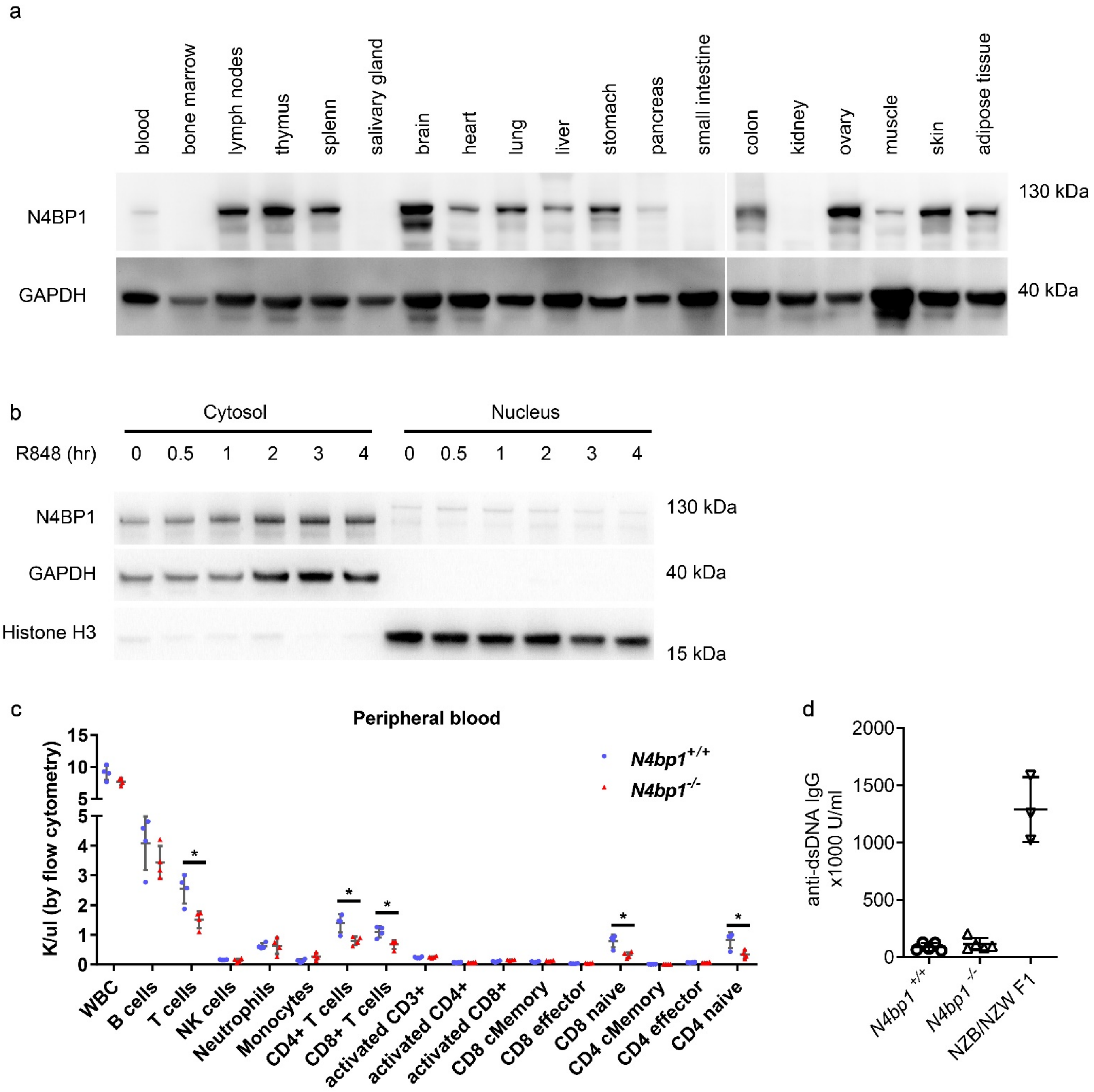

Supplementary Figure 2. N4BP1-expressing tissues, and immune cell populations in peripheral blood and dsDNA antibodies in *N4bp1*$^{-/-}$ mice. a, b, Immunoblot analysis of N4BP1 expression in different tissues (**a**) or cellular compartments of peritoneal macrophages (**b**). **c,** Quantification of lymphoid and myeloid cells analyzed by flow cytometry in the peripheral blood of wild-type and *N4bp1*$^{-/-}$ mice (n=4 mice per genotype). **P*=0.0104, **P*=0.0125, **P*=0.0113, **P*=0.0101, **P*=0.0177, unpaired, two-tailed Student's *t*-test. **d,** Serum concentration of anti-dsDNA in wild-type and *N4bp1*$^{-/-}$ mice (n=5 mice per genotype). NZB/NZW F1 mice were used as positive controls (n=3 mice). Each symbol (**c,d**) represents an individual mouse. Data are representative of two independent experiments (**a-d**). Mean ± s.d. plotted in **c,d**. *P* values are listed in order from left to right. Source data are provided in the Source Data file.

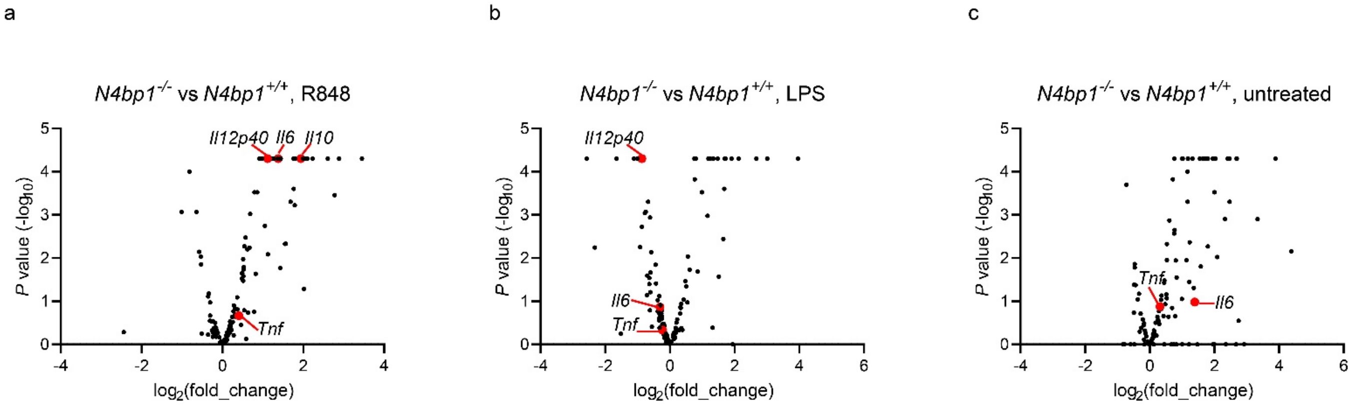

Supplementary Figure 3. RNAseq for *N4bp1*$^{+/+}$ and *N4bp1*$^{-/-}$ peritoneal macrophages. a-c, Comparative analysis of expression levels of 124 NF-κB-dependent genes measured by RNA sequencing in *N4bp1*$^{+/+}$ vs. *N4bp1*$^{-/-}$ peritoneal macrophages stimulated with R848 (**a**), LPS (**b**) or without any stimulation (**c**). Cells were collected from two mice for each condition. Experiment was performed once. Source data are provided in the Source Data file.

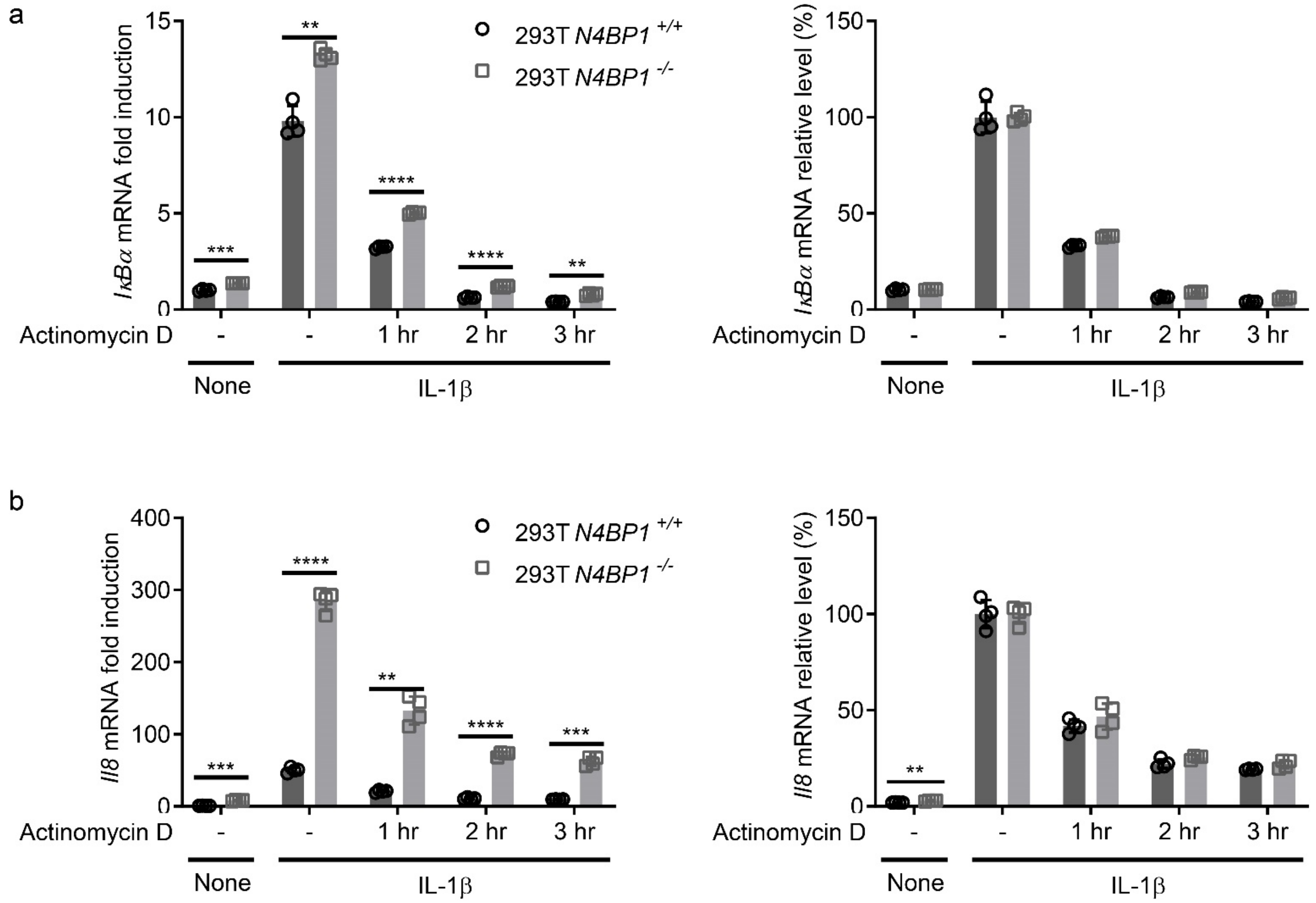

Supplementary Figure 4. Comparable mRNA degradation rates in wild-type and *N4BP1*$^{-/-}$ HEK293T cells. a, b, RT-qPCR analysis of *NFKBIA* (*IκBα*) (**a**) or *IL8* (**b**) in wild-type and *N4BP1*$^{-/-}$ HEK293T stimulated with IL-1β for 2 h then treated without or with actinomycin D (2 μg/ml) for the indicated times. *Left*, mRNA fold induction. *Right*, amount of mRNA relative to that of cells stimulated with IL-1β but without Actinomycin D, set as 100%. ***P=0.0004, **P=0.0017, ****P<0.0001, ****P<0.0001, **P=0.0014 (**a**), ***P=0.0002, ****P<0.0001, **P=0.0013, ****P<0.0001, ***P=0.0004 [left], **P=0.0012 [right] (**b**), unpaired, two-tailed Student's *t*-test. Data points represent independent cultures (n=4 independent treatments of 4 cultures split from one cell line). Data are representative of two independent experiments (mean ± s.d.). *P* values are listed in order from left to right. Source data are provided in the Source Data file.

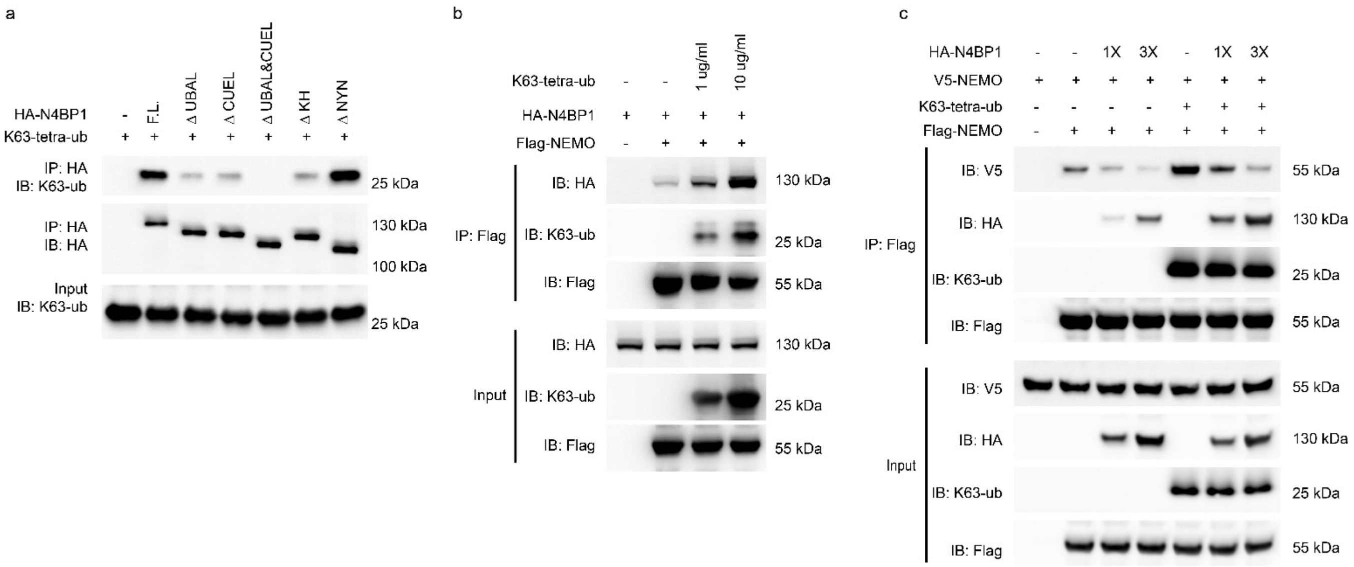

Supplementary Figure 5. K63-linked ubiquitin enhances N4BP1 and NEMO interaction. a, K63-linked ubiquitin was incubated without (-) or with purified recombinant HA-N4BP1 or its domain deletion forms and subjected to immunoprecipitation with anti-HA. **b,** Purified recombinant HA-N4BP1 and Flag-NEMO were incubated without (-) or with K63-linked ubiquitin as indicated, and subjected to immunoprecipitation with anti-Flag. **c,** Purified recombinant Flag-NEMO and V5-NEMO were mixed together and then incubated with different amounts of HA-N4BP1 in the presence of K63-linked ubiquitin or without ubiquitin, and subjected to immunoprecipitation with anti-Flag. Complexes were analyzed by immunoblotting with the indicated antibodies (**a-c**). Data are representative of two independent experiments. Source data are provided in the Source Data file.

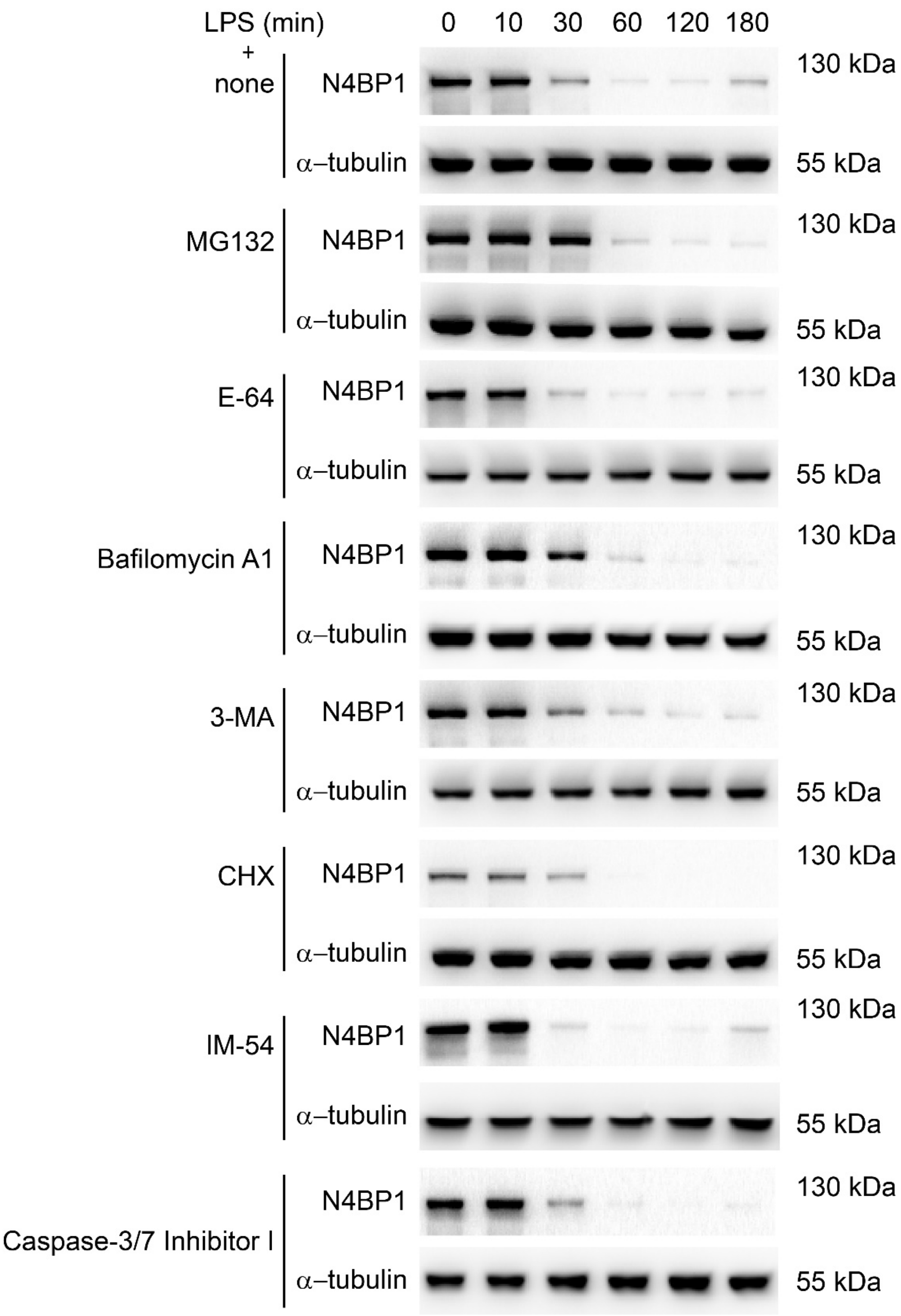

Supplementary Figure 6. Downregulation of N4BP1 is not mediated by the proteasome, autophagy, or cysteine proteases. Immunoblot analysis of N4BP1 in peritoneal macrophages pretreated with MG132 (10 μM), E-64 (20 μM), bafilomycin A1 (1 μM), 3-MA (5 mM), IM-54 (50 μM), or caspase-3/7 Inhibitor I (100 μM) for 1 h, or with CHX (50 μg/ml) for 3 h then stimulated with LPS for the indicated times. Data are representative of two independent experiments. Source data are provided in the Source Data file.

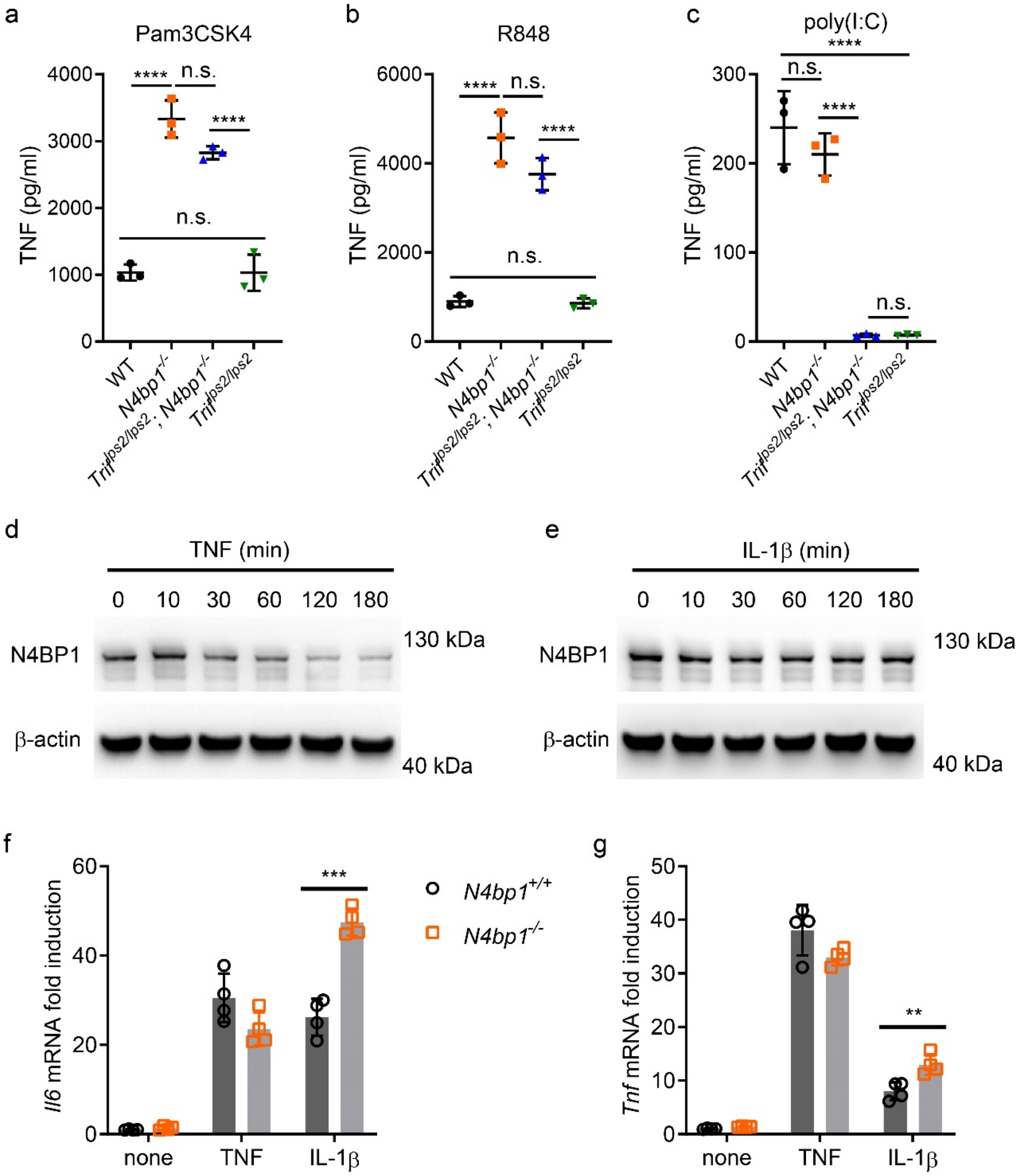

Supplementary Figure 7. *N4bp1* deficiency enhanced IL-1β but not TNF signaling. a-c, TNF concentration in the culture medium of wild-type (WT), *N4bp1*$^{-/-}$, *Trif*$^{lps2/lps2}$*N4bp1*$^{-/-}$, and *Trif*$^{lps2/lps2}$ peritoneal macrophages treated with Pam3CSK4 (**a**), R848 (**b**), or poly(I:C) (**c**) (n=3 mice per genotype). ****P<0.0001, one-way ANOVA and post hoc Tukey test. **d, e,** Immunoblot analysis of N4BP1 in peritoneal macrophages treated with TNF (100 ng/ml) (**d**) or IL-1β (100 ng/ml) (**e**). **f, g,** RT-qPCR analysis of *Il6* (***P=0.0003, unpaired, two-tailed Student's *t*-test) (**f**) or *Tnf* (**P=0.0082, unpaired, two-tailed Student's *t*-test) (**g**) in *N4bp1*$^{+/+}$ and *N4bp1*$^{-/-}$ peritoneal macrophages stimulated with TNF or IL-1β for 2 h (n=4 mice per genotype). Each symbol represents an individual mouse; equal numbers of macrophages from each mouse were treated as indicated (**a-c, f, g**). Data are representative of two independent experiments (**a-g**). Mean ± s.d. plotted in **a-c, f, g**. Source data are provided in the Source Data file.

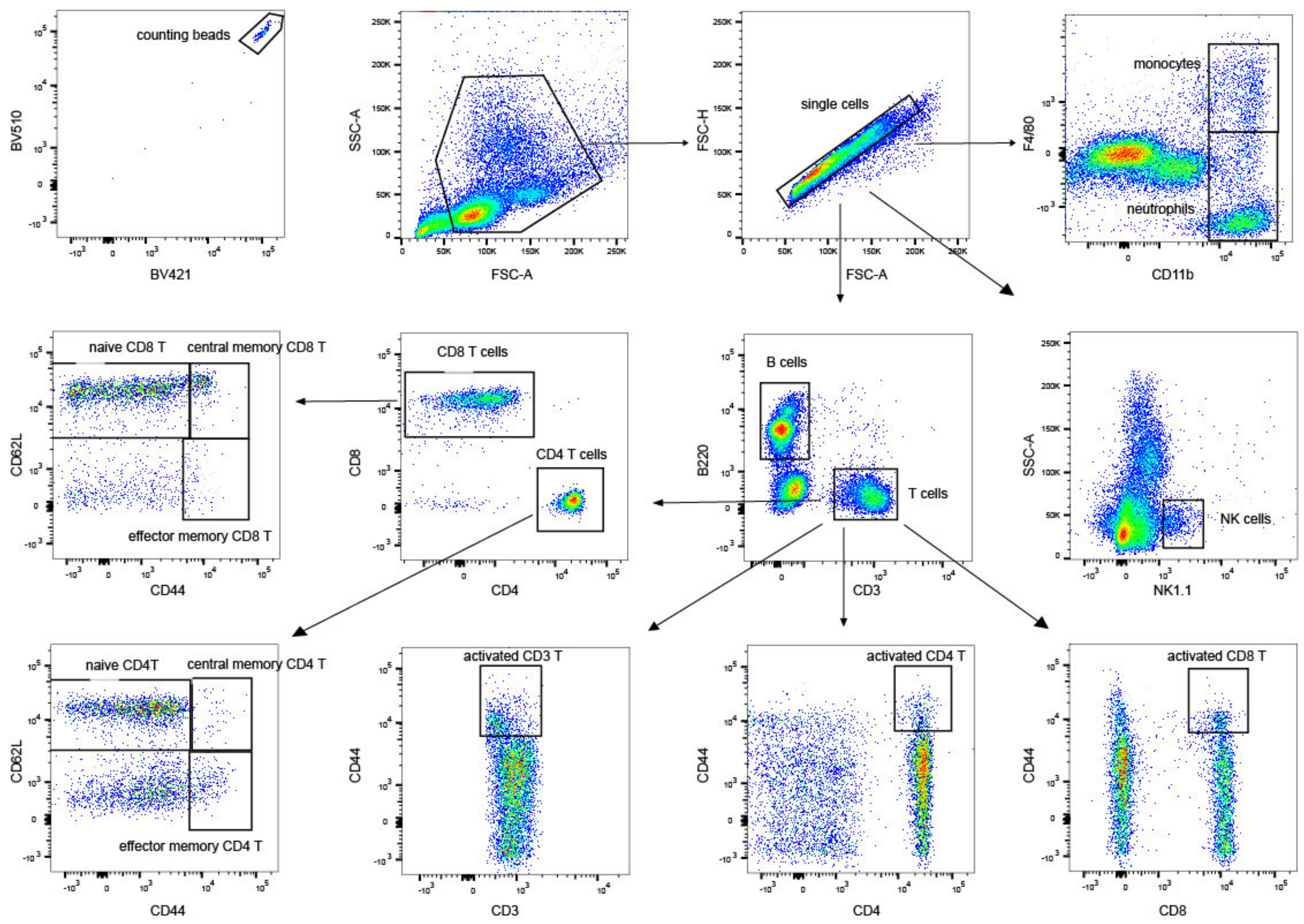

Supplementary Figure 8. Gating strategies for flow cytometric analysis of immune cells in the blood. These were used to obtain data shown in Supplementary Fig. 2c.

Supplementary Table 1. Primer sequences

Name	Sequence (5’ to 3’)
Genotyping	
N4bp1 KO allele.PCR1	CTGGTGAATTGGTCTAACTTTGTC
N4bp1 KO allele.PCR2	AAACTGCTGAATGTGACTCC
N4bp1 KO allele.sequencing	AAACTGCTGAATGTGACTCC
ChIP	
Il6 promoter.F	AATGTGGGATTTTCCCATGA
Il6 promoter.R	GCTCCAGAGCAGAATGAGCTA
qPCR	
Il1β.F	TGTAATGAAAGACGGCACACC
Il1β.R	TCTTCTTTGGGTATTGCTTGG
Il6.F	CTCTGCAAGAGACTTCCATCC
Il6.R	CGACTTGTGAAGTGGTATAGACAG
Il10.F	TGGCCCAGAAATCAAGGAGC
Il10.R	CAGCAGACTCAATACACACT
Il12p40.F	GGAAGCACGGCAGCAGAATA
Il12p40.R	AACTTGAGGGAGAAGTAGGAATGG
Tnf.F	CTGTAGCCCACGTCGTAGC
Tnf.R	TTGAGATCCATGCCGTTG
Ccl5.F	ACTCCCTGCTGCTTTGCCTAC
Ccl5.R	TGCTGCTGGTGTAGAAATACT
Gapdh.F	CGTCCCGTAGACAAAATGGT
Gapdh.R	TTGATGGCAACAATCTCCAC
huGAPDH.F	ACCCACTCCTCCACCTTTGA
huGAPDH.R	CTGTTGCTGTAGCCAAATTCGT
huNfkbia.F (IκBα)	CTCCGAGACTTTCGAGGAAATAC
huNfkbia.R (IκBα)	GCCATTGTAGTTGGTAGCCTTCA
huIL8.F	ATAAAGACATACTCCAAACCTTTCCAC
huIL8.R	AAGCTTTACAATAATTTCTGTGTTGGC
