# Supplementary material for: N4BP1 negatively regulates NF-κB by binding and inhibiting NEMO oligomerization
Source: Nat Commun. 2021 Mar 2;12:1379. doi: 10.1038/s41467-021-21711-5 (PMC7925594; doi:10.1038/s41467-021-21711-5)
Supplement: Supplementary file 3 — Descriptions of Additional Supplementary Files [file 41467_2021_21711_MOESM3_ESM.pdf]

**Descriptions of Additional Supplementary Files**

**Supplementary Data 1**

**Description**: RNAseq analysis of NF-κB-dependent genes in N4bp1+/+ and N4bp1-/- peritoneal macrophages
