# Supplementary material for: N4BP1 negatively regulates NF-κB by binding and inhibiting NEMO oligomerization
Source: Nat Commun. 2021 Mar 2;12:1379. doi: 10.1038/s41467-021-21711-5 (PMC7925594; doi:10.1038/s41467-021-21711-5)
Supplement: Supplementary file 5 — Reporting Summary [file 41467_2021_21711_MOESM5_ESM.pdf]

nature research

Corresponding author(s): Bruce Beutler

Last updated by author(s): Jan 20, 2021

Reporting Summary

Nature Research wishes to improve the reproducibility of the work that we publish. This form provides structure for consistency and transparency in reporting. For further information on Nature Research policies, see our Editorial Policies and the Editorial Policy Checklist.

Statistics

For all statistical analyses, confirm that the following items are present in the figure legend, table legend, main text, or Methods section.

n/a	Confirmed	
☐	☒	The exact sample size (n) for each experimental group/condition, given as a discrete number and unit of measurement
☐	☒	A statement on whether measurements were taken from distinct samples or whether the same sample was measured repeatedly
☐	☒	The statistical test(s) used AND whether they are one- or two-sided<br>*Only common tests should be described solely by name; describe more complex techniques in the Methods section.*
☒	☐	A description of all covariates tested
☐	☒	A description of any assumptions or corrections, such as tests of normality and adjustment for multiple comparisons
☐	☒	A full description of the statistical parameters including central tendency (e.g. means) or other basic estimates (e.g. regression coefficient) AND variation (e.g. standard deviation) or associated estimates of uncertainty (e.g. confidence intervals)
☐	☒	For null hypothesis testing, the test statistic (e.g. F, t, r) with confidence intervals, effect sizes, degrees of freedom and P value noted<br>*Give P values as exact values whenever suitable.*
☒	☐	For Bayesian analysis, information on the choice of priors and Markov chain Monte Carlo settings
☒	☐	For hierarchical and complex designs, identification of the appropriate level for tests and full reporting of outcomes
☒	☐	Estimates of effect sizes (e.g. Cohen's d, Pearson's r), indicating how they were calculated

Our web collection on statistics for biologists contains articles on many of the points above.

Software and code

Policy information about availability of computer code

Data collection	Flow cytometry data were acquired on a BD LSRFortessa (BD Biosciences). RNA sequencing was performed using an Illumina HiSeq 2500.
Data analysis	Flow cytometry data were analyzed using FlowJo software. GraphPad Prism software was used to prepare graphs and to perform statistical analyses. For RNA sequencing analysis, CASAVA, tophat (v. 2.0.10), cufflinks, and cuffmerge (v.2.1.1) software was used.

For manuscripts utilizing custom algorithms or software that are central to the research but not yet described in published literature, software must be made available to editors and reviewers. We strongly encourage code deposition in a community repository (e.g. GitHub). See the Nature Research guidelines for submitting code & software for further information.

Data

Policy information about availability of data

All manuscripts must include a data availability statement. This statement should provide the following information, where applicable:

- Accession codes, unique identifiers, or web links for publicly available datasets
- A list of figures that have associated raw data
- A description of any restrictions on data availability

All data are available in the main text or the supplementary information.

Field-specific reporting

Life sciences study design

All studies must disclose on these points even when the disclosure is negative.

Sample size	No pre-specified effect size was assumed, and in general three or more animals or replicates for each genotype or condition were used in experiments; this sample size was sufficient to demonstrate statistically significant differences in comparisons between two unpaired experimental groups by unpaired t-test.
Data exclusions	No data were excluded from any analyses.
Replication	The RNA sequencing experiment was performed one time. All other experiments were repeated at least twice and all attempts at replication were successful.
Randomization	The allocation of samples or animals to experimental groups was random.
Blinding	The investigator was not blinded to genotypes or group allocations during any experiment.

Reporting for specific materials, systems and methods

We require information from authors about some types of materials, experimental systems and methods used in many studies. Here, indicate whether each material, system or method listed is relevant to your study. If you are not sure if a list item applies to your research, read the appropriate section before selecting a response.

Materials & experimental systems

n/a	Involved in the study	
☐	☒	Antibodies
☐	☒	Eukaryotic cell lines
☒	☐	Palaeontology and archaeology
☐	☒	Animals and other organisms
☒	☐	Human research participants
☒	☐	Clinical data
☒	☐	Dual use research of concern

Methods

n/a	Involved in the study	
☒	☐	ChIP-seq
☐	☒	Flow cytometry
☒	☐	MRI-based neuroimaging

Antibodies

Antibodies used	Antibodies to the following were used: mouse N4BP1 (EPNCIR118, 1:1000), human N4BP1 (ab209103, 1:1000) and NEMO (EPR16629, 1:1000) (Abcam); HA (6E2, HRP conjugate, 1:1000), GAPDH (8884, 1:2000), p65 (D14E12, 1:1000), p-p65 (93H1, 1:1000), p-IKKα/β (16A6, 1:1000), IκBα (L35A5, 1:1000), p-p38 (4511, 1:1000), p-c-Jun (D47G9, 1:1000), Histone H3 (9715, 1:2000), and GAPDH (D16H11, 1:2000) (Cell Signaling Technology); Flag (M2, 1:5000), V5 (V8012, 1:2000), β-actin (A2228, 1:2000) and α-tubulin (T6199, 1:2000) (Sigma-Aldrich); Acetyl-Histone H4 (PA5-40084, 1:1000, Invitrogen); Linear ubiquitin (LUB9, 1:1000, Millipore); K63-polyubiquitin (BML-PW0600, 1:1000, ENZO Life Sciences). Primary antibodies used for flow cytometry were: CD3ε (clone 145-2C11, 1:100), CD4 (clone RM4-5, 1:200), CD8α (clone 53-6.7, 1:200), B220 (CD45R, clone RA3-6B2, 1:200), NK-1.1 (clone PK136, 1:200), CD44 (clone IM7, 1:300), CD62L (clone MEL-14, 1:300), CD11c (clone HL3, 1:200), CD11b (clone M1/70, 1:200), F4/80 (clone BM8, 1:200), CD16/32 (Clone 2.4G2, 1:200) (BD Biosciences).
Validation	All antibodies used in this study were previously validated for the specific application by the companies from which we purchased them.

Eukaryotic cell lines

Policy information about cell lines

Cell line source(s)	HEK293T and THP-1 cells were purchased from ATCC.
Authentication	All cell lines from ATCC have been thoroughly tested and authenticated by ATCC.
Mycoplasma contamination	The cell lines from ATCC were directly used in experiments and were not tested for mycoplasma contamination.
Commonly misidentified lines (See ICLAC register)	This study did not involve commonly misidentified lines.

Animals and other organisms

Policy information about studies involving animals; ARRIVE guidelines recommended for reporting animal research

Laboratory animals	Eight- to twelve-week old male and female mice (Mus musculus) on a pure C57BL/6J background were used in experiments.
Wild animals	This study did not involve wild animals.
Field-collected samples	This study did not involve field-collected samples.
Ethics oversight	All experimental procedures using mice were approved by the Institutional Animal Care and Use Committee of the University of Texas Southwestern Medical Center and were conducted in accordance with institutionally approved protocols and guidelines for animal care and use.

Note that full information on the approval of the study protocol must also be provided in the manuscript.

Flow Cytometry

Plots

Confirm that:

- [ ] The axis labels state the marker and fluorochrome used (e.g. CD4-FITC).
- [x] The axis scales are clearly visible. Include numbers along axes only for bottom left plot of group (a 'group' is an analysis of identical markers).
- [x] All plots are contour plots with outliers or pseudocolor plots.
- [x] A numerical value for number of cells or percentage (with statistics) is provided.

Methodology

Sample preparation	Blood was collected in Minicollect Tubes (Mercedes Medical) and centrifuged at 700g for separation of serum, and red blood cells remaining in the serum were lysed using RBC lysis buffer (eBioscience) before staining of immune cells and flow cytometry analysis. Cells were incubated with monoclonal antibody to CD16/32 and were labeled for 1 h at 4 °C using fluorochrome-conjugated monoclonal antibodies to mouse CD3ε, CD4, CD8α, CD44, CD62L, B220, NK1.1, CD11b, F4/80 and CD11c. Counting beads (Invitrogen) were added to the samples before collecting data.
Instrument	BD LSRFortessa (BD Biosciences)
Software	Flow cytometry data were analyzed using FlowJo software.
Cell population abundance	The abundance of relevant cell populations in the peripheral blood of mice was significant to gate and calculate, and was within a reasonable range. The purity of cell populations was determined using commercially available monoclonal antibodies against the specific surface markers on each cell type.
Gating strategy	Counting beads were gated by two free channels. The cell populations were gated as illustrated in Supplementary Fig. 8. Monocytes (CD11b high, F4/80 high); neutrophils (CD11b high, F4/80 low); NK cells (NK1.1 positive); B cells (B220 high, CD3 negative); T cells (CD3 high, B220 low); CD4 T (CD3, CD4 high, CD8, B220 negative); CD8 T (CD3, CD8 high, CD4, B220 negative); activated T cells (CD3, CD44 high, B220 negative); activated CD4 T cells (CD3, CD4, CD44 high, B220 negative); activated CD8 T cells (CD3, CD8, CD44 high, B220 negative); naive T cells (CD4 or CD8 positive, CD3, CD62L high, CD44, B220 low); central memory T cells (CD4 or CD8 positive, CD3, CD62L, CD44 high, B220 low); effector memory T cells (CD4 or CD8 positive, CD3, CD44 high, CD62L, B220 low).

- [x] Tick this box to confirm that a figure exemplifying the gating strategy is provided in the Supplementary Information.
