# Supplementary material for: N4BP1 negatively regulates NF-κB by binding and inhibiting NEMO oligomerization
Source: Nat Commun. 2021 Mar 2;12:1379. doi: 10.1038/s41467-021-21711-5 (PMC7925594; doi:10.1038/s41467-021-21711-5)
Supplement: Supplementary file 2 — Peer Review File [file 41467_2021_21711_MOESM2_ESM.pdf]

Editorial Note: This manuscript has been previously reviewed at another journal that is not operating a transparent peer review scheme. This document only contains reviewer comments and rebuttal letters for versions considered at Nature Communications.

REVIEWERS' COMMENTS

Reviewer #1 (Remarks to the Author):

The authors have done a thorough job addressing my concerns.

Reviewer #2 (Remarks to the Author):

In the revised version of their manuscript, the authors have adequately and satisfactorily addressed all points raised in my review of the previous version of this manuscript.

Reviewer #3 (Remarks to the Author):

The authors have responded sufficiently to all my concerns. I support publication of this manuscript in Nature Communications.

The authors should clarify in the Figure legend to Supplementary Fig. 1A-D what statistical test was used for these panels.
